# Prevalence and associated risk factors of asymptomatic malaria in Nigeria: a systematic review and meta-analysis

**DOI:** 10.1186/s12936-025-05671-5

**Published:** 2025-12-31

**Authors:** Alameen Mukhtar, Mubarak Ismail, Aminu Usman, Ismail Ayoade Odetokun, Mohammed Auwal Ibrahim, Abdulmalik Abdullahi Salman, Hafsatu Garba Bawa-Sani, Shafique Sani Nass, Baba Waru Goni, Muhammad Nazir Shehu, Abdulkadir Nuhu, Xiaoying Zhang, Murtala Bindawa Isah

**Affiliations:** 1 Joint Laboratory for Antibody Research and Application, Shaanxi International Cooperation Demonstration Base, Shaanxi University of Technology, Hanzhong, 723000 Shaanxi China; 2 Department of Biochemistry, Umaru Musa Yar’adua University , Katsina, Nigeria; 3 Biomedical Science Research and Training Centre, Yobe State University, Damaturu, Yobe State Nigeria; 4 Department of Veterinary Public Health and Preventive Medicine, University of Ilorin, Ilorin, Nigeria; 5 Department of Biochemistry, Ahmadu Bello University, Zaria, Nigeria; 6 Faculty of Basic Clinical Sciences, Umaru Musa Yar’adua University, Katsina, Nigeria; 7 National Open University of Nigeria, Abuja, Nigeria; 8 Federal Teaching Hospital Katsina, Katsina, Nigeria

**Keywords:** Asymptomatic malaria, Nigeria, Prevalence, Risk factors, Children, Pregnant women, Malaria control

## Abstract

**Background:**

Asymptomatic malaria presents a significant barrier to malaria elimination efforts, particularly in endemic countries like Nigeria. Despite its public health relevance, no national-level pooled estimate of its prevalence and associated risk factors currently exists for Nigeria. This systematic review and meta-analysis aimed to synthesize existing data to estimate the prevalence of asymptomatic malaria and identify affected populations and risk factors across Nigeria.

**Methods:**

A systematic search of PubMed, Google Scholar, and Scopus was conducted to identify observational studies reporting the prevalence of asymptomatic malaria in Nigeria. The review protocol was registered with PROSPERO (CRD42024591788). Eligible studies were screened using Rayyan software, and relevant data were extracted into Microsoft Excel. Meta-analysis was performed using Stata version 15.0. A random-effects model was applied to estimate the pooled prevalence. Heterogeneity was assessed using meta-regression and subgroup analyses, while publication bias was evaluated using funnel plot visualization.

**Results:**

A total of 25 studies were included in the meta-analysis. The pooled prevalence of asymptomatic malaria in Nigeria was estimated at 33% (95% CI 26–41). Prevalence varied by population subgroup, ranging from 25% in the general population to 52% in children. Risk factor analysis revealed an overall prevalence of 19% related to education level, 36% based on sex, and 39% associated with insecticide-treated net (ITN) usage.

**Conclusion:**

This review highlights a high prevalence of asymptomatic malaria in Nigeria, particularly among children. While asymptomatic infections sustain transmission, current evidence underscores the need to prioritize proven transmission-reduction tools. With reduced transmission, the asymptomatic reservoir can then be more effectively addressed through complementary strategies.

**Supplementary Information:**

The online version contains supplementary material available at 10.1186/s12936-025-05671-5.

## Background

Malaria is a parasitic infection of human erythrocytes caused by protozoan parasites of the genus *Plasmodium*. Transmission occurs through the bite of infected female *Anopheles* mosquitoes [1]. Despite nearly seven decades since the first global eradication initiative, malaria continues to impose a substantial burden of mortality and morbidity, particularly among children in low- and middle-income countries of sub-Saharan Africa.

In 2022, the World Health Organization (WHO) reported 249 million malaria cases across 85 countries, resulting in approximately 600,000 deaths, predominantly among children [2]. The global burden remains highly concentrated, with approximately 70% of cases occurring in just 11 countries, ten of which are located in sub-Saharan Africa. Nigeria bears the heaviest malaria burden globally, with an estimated 68 million cases and 194,000 deaths recorded in 2021, representing more than 25% of the global malaria burden. The country alone contributes one-quarter of all malaria cases among the 45 malaria-endemic African nations [3]. Children under 5 years of age are disproportionately affected, with malaria accounting for 20% of all deaths in this age group in 2021 [4].

The global malaria elimination agenda has achieved success in 12 countries over the past decade. Current elimination strategies in Nigeria and across Africa include artemisinin-based combination therapy (ACT), which represents the most effective antimalarial treatment regimen available. However, several critical challenges threaten ongoing elimination efforts. Artemisinin resistance has been documented in three of the five human *Plasmodium* species [5]. Nigeria’s malaria control efforts face additional obstacles, including drug and insecticide resistance, inadequate surveillance systems, and insufficient healthcare infrastructure, which collectively impede effective case management, vector control, and disease prevention [4]. Among these challenges, asymptomatic malaria represents one of the most critical yet frequently overlooked barriers to elimination [6].

Individuals harbouring *Plasmodium* parasites without manifesting clinical symptoms function as cryptic reservoirs that sustain transmission and complicate eradication efforts [7]. These Undetected asymptomatic infections pose a major challenge for malaria control, as they evade conventional diagnostic methods and sustain transmission. In low and very low transmission settings, the World Health Organization in 2018 recommends targeted surveillance strategies such as active case detection, though their utility remains limited to these contexts [8]. Asymptomatic malaria is characterized by the absence of clinical manifestations despite parasitaemia. This condition is predominantly associated with partial immunity acquired through repeated parasite exposure, making it more prevalent among specific populations, particularly children and pregnant women. In children less than < 5 years, immature immune systems increase illness susceptibility, while pregnant women face elevated risks due to physiological changes that predispose them to adverse outcomes, including anaemia and low birth weight. Following infection, *Plasmodium* parasite densities frequently remain extremely low and often undetectable through routine microscopy. Although most infected adults remain clinically asymptomatic, they maintain the capacity for transmission through persistent low-density gametocytaemia [9]. Asymptomatic malaria can occur across all age groups [10]. Beyond serving as transmission reservoirs, asymptomatic malaria contributes to various clinical conditions, including quartan nephropathy (a cause of nephrotic syndrome in tropical children resulting from subclinical *Plasmodium malariae* infection), tropical splenomegaly syndrome (hyperreactive malarial splenomegaly causing massive splenomegaly), and Burkitt’s lymphoma in children [11].

Detection of asymptomatic malaria presents significant challenges due to extremely low parasite densities that render microscopic detection unreliable, necessitating more sensitive molecular methods such as polymerase chain reaction (PCR) [12]. In Nigeria, advanced molecular diagnostics are not routinely employed despite their superior sensitivity and accuracy, with PCR detecting approximately twice as many infections as microscopy [13]. Beyond diagnostic limitations, however, the greater challenge lies in identifying asymptomatic individuals, since they do not seek care in the absence of illness. Detecting such infections typically requires population-based surveys (e.g., Demographic and Health Surveys (DHS), Malaria Indicator Surveys (MIS)) or specialized screening approaches, both of which demand substantial financial and programmatic resources. PCR implementation faces substantial barriers, including high equipment and reagent costs, inadequate laboratory infrastructure, and scarcity of trained personnel. These limitations are particularly pronounced in rural and underserved areas where asymptomatic malaria prevalence is highest. In low-transmission settings, submicroscopic infections constitute 70–80% of infections, yet remain undetected by standard microscopy. The limited access to advanced diagnostics, combined with financial constraints and insufficient human resources, results in widespread underdiagnoses of asymptomatic malaria, perpetuating transmission and undermining control efforts. Moreover, asymptomatic malaria becomes a primary control concern during the transition from malaria control to elimination, when the hidden reservoir of asymptomatic infections becomes the dominant source of ongoing transmission, typically occurring after a significant reduction in clinical cases has been achieved, but local transmission persists [9].

Global malaria elimination strategies aim for zero transmission; however, their operational focus varies depending on the intensity of transmission. In high-transmission settings such as sub-Saharan Africa, including Nigeria, most malaria infections are asymptomatic at the time of detection, acting as a critical parasite reservoir that sustains ongoing transmission [13]. National surveys such as the DHS and MIS, alongside community-based studies, have consistently documented high asymptomatic malaria prevalence across the region [14–16]. Despite its relevance to malaria control, the prevalence and risk factors of asymptomatic malaria in Nigeria have not been systematically assessed. Synthesizing this evidence is crucial for informing evidence-based policy decisions and developing effective strategic interventions.

Therefore, a systematic review and meta-analysis of asymptomatic malaria in Nigeria, the country with the highest global malaria burden, was conducted to provide comprehensive evidence for strengthening elimination strategies.

### Search strategy

This systematic review and meta-analysis were conducted following the Preferred Reporting Items for Systematic Reviews and Meta-Analyses (PRISMA) guidelines [17]. A comprehensive literature search was performed on Monday, 23 August 2024**,** across three electronic databases: PubMed**,** Google Scholar**,** and Scopus**.** The search aimed to identify studies investigating asymptomatic malaria in Nigeria**,** using combinations of the keywords: “asymptomatic malaria,” “carrier state,” “prevalence,” and “Nigeria.” To ensure originality and avoid duplication, existing reviews on the subject were checked in advance.

This review protocol was registered with the International Prospective Register of Systematic Reviews (PROSPERO) under the registration number CRD42024591788. The review addressed two primary outcomes: first, to estimate the prevalence of asymptomatic malaria in Nigeria, and second, to identify the most affected study population, whether the general population, pregnant women, adults, or children, and to explore associated risk factors contributing to asymptomatic malaria.

### Selection and eligibility criteria

*Inclusion criteria*: The studies included are cross-sectional studies conducted on the prevalence of asymptomatic malaria in Nigeria. The included studies must provide essential information about sample size, asymptomatic malaria prevalence, diagnostic methods (rapid diagnostic test (RDT), PCR, or microscopy), study populations (adults, children (≤ 15 years), pregnant women, and the general population), and study areas. All included studies were original research published in English, with year of publication ranging from 2005 to 2024. Participants must not have exhibited any symptoms of malaria.

*Exclusion criteria*: Studies published in a language other than English were excluded, as were posters, conference papers, and review papers. Studies without available full texts were also excluded, as well as studies that did not specify the conditions of the participants.

### Data screening, quality assessment and data extraction

The study utilized a two-step screening process, comprising primary and secondary screening. Two independent assessors (A.M and M.I.) conducted the initial screening of titles and abstracts using Rayyan software [18] for duplicate removal and inclusion evaluation. Discrepancies identified during this phase were resolved by consensus, or when unresolved, through consultation with a third reviewer. The secondary screening involved a full-text review to ensure adherence to inclusion criteria, employing the same resolution process for discrepancies. The Newcastle–Ottawa Scale (NOS), adapted for cross-sectional studies, was applied to assess the methodological quality of included studies (Tables S1 and S2). This scale evaluated aspects such as sample selection, comparability of groups, and ascertainment of outcomes to ensure robust quality standards.

Data extraction was performed using a structured sheet in Microsoft Excel. Key study characteristics were recorded, including the year of publication, sample size, author name, study design, study population, geographic location, diagnostic methods, and the prevalence of asymptomatic malaria (Table S3). This systematic approach ensured uniformity and accuracy across the dataset.

### Statistical analysis

All extracted data were organized in a Microsoft Excel spreadsheet and subsequently imported into Stata statistical software (version 15.0) for meta-analysis. A random-effects model, based on the Der Simonian and Laird method, was employed to estimate the pooled prevalence of asymptomatic malaria, along with the corresponding 95% confidence intervals. To assess the degree of heterogeneity among the included studies, both the chi-square test and the I^2^ statistic were calculated. The I^2^ statistic quantifies the proportion of variation across studies that is due to heterogeneity rather than chance, with values greater than 75% considered to indicate substantial heterogeneity. To further explore potential sources of variation, subgroup analyses were conducted according to study population, geographic region, and diagnostic method, using the “*metan*” command in Stata.

## Results

### Literature search result

**Fig. 1:**
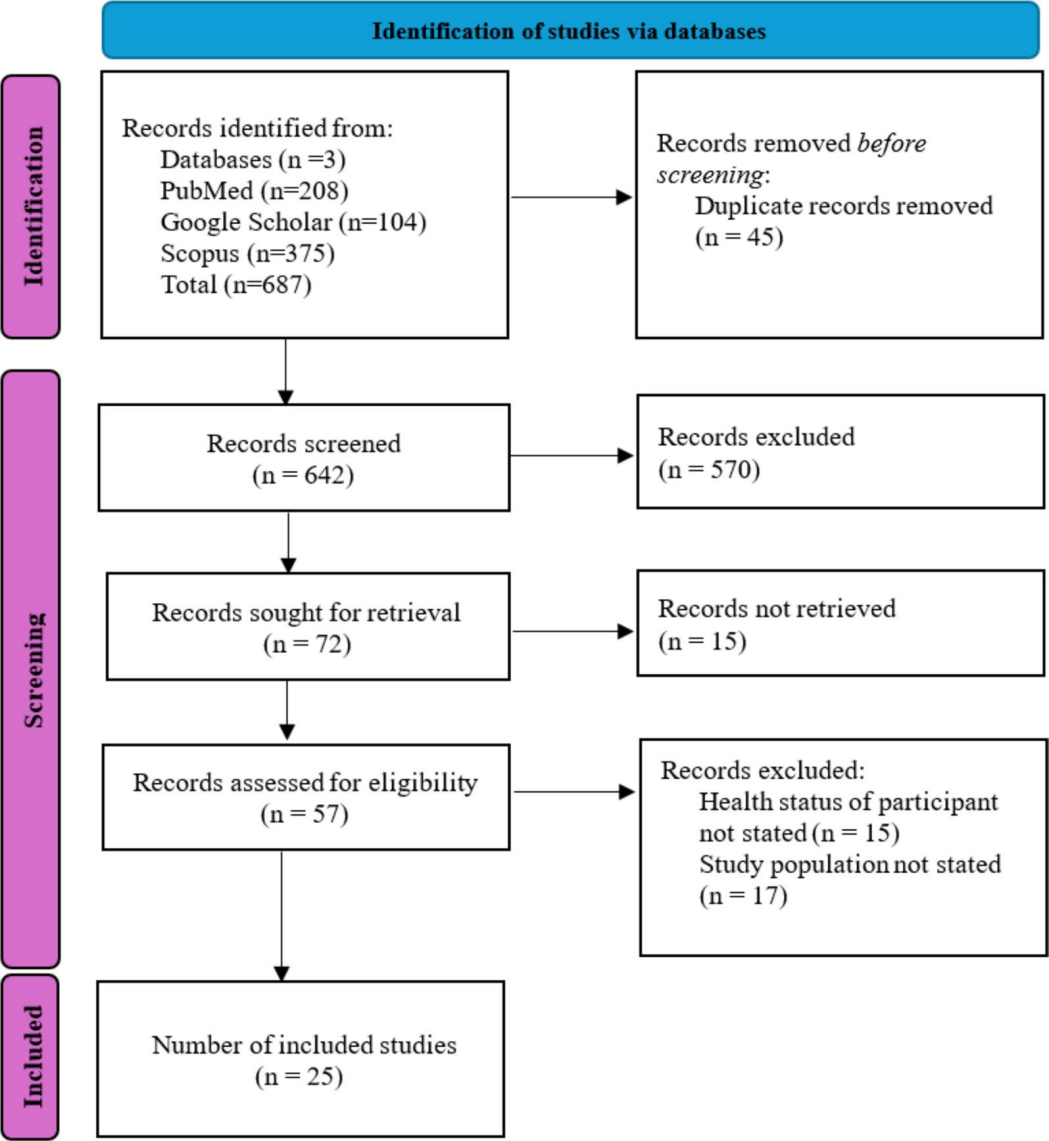
PRISMA flow diagram showing the systematic literature search and study selection process for the studies on asymptomatic malaria prevalence in Nigeria. The diagram illustrates the identification, screening, eligibility assessment, and inclusion phases of the systematic review. Initial searches across four databases (PubMed, Google Scholar, Scopus, and additional databases) yielded 687 records. After removing 45 duplicate records, 642 records underwent title and abstract screening. Following full-text assessment of 57 potentially eligible studies, 25 studies met the inclusion criteria and were included in the final meta-analysis. The main reasons for exclusion were failure to report participant health status (n = 15) and unspecified study population (n = 17). PRISMA, Preferred Reporting Items for Systematic Reviews and Meta-Analyses

A total of 687 published articles were initially retrieved through searches across the selected electronic databases. Following the removal of 45 duplicate records, 642 unique articles remained for screening. Titles and abstracts of these articles were reviewed, resulting in the exclusion of 570 studies that did not meet the inclusion criteria. The remaining 72 articles underwent full-text assessment to determine their eligibility. Of these, 15 were excluded due to unavailable full texts, while 32 did not satisfy the predefined eligibility criteria. Ultimately, 25 studies met all inclusion requirements and were included in both the qualitative synthesis and the meta-analysis. The selection process is illustrated in the PRISMA flowchart presented in Fig. 1.

### Description of included studies

Twenty-five studies met the inclusion criteria and were included in this systematic review. The studies encompassed diverse populations: nine studies (36%) focused on pregnant women, five studies (20%) on children primarily under 5 years, though two included broader age ranges (3–15 years), five studies (20%) on adults, and six studies (24%) examined the general population (Table 1). The combined sample size across all studies was 7956 participants, with 2760 positive cases of asymptomatic malaria identified.

**Table 1:** The pooled prevalence of asymptomatic malaria in Nigeria and Subgroup Analysis

Variables	Number of Studies	Sample size	Positive Cases	Prevalence (95% Confidence interval)	Heterogeneity ^(%)^	*P–*value
Overall prevalence	25	7956	2760	0.33 (0.26; 0.41)	98.6%	< 0.001
Study population						
General Population	6	2735	631	0.25 (0.19; 0.32)	93.5%	< 0.001
Pregnant women	9	2127	550	0.27 (0.16; 0.38)	97.8%	< 0.001
Adult	5	1974	982	0.37 (0.16; 0.58)	98.8%	< 0.001
Children	5	1120	597	0.52 (0.41; 0.52)	91.5%	< 0.001
Regions						
Southern	19	5428	1682	0.33 (0.26; 0.41)	98%	< 0.001
Northern	6	2528	1078	0.33 (0.13; 0.53)	99.3%	< 0.001
Diagnostic method						
Microscopy	15	4373	1457	0.35 (0.26; 0.45)	96.3%	< 0.001
RDT	3	1623	868	0.41 (0.19; 0.63)	96.2%	< 0.001
Microscopy and PCR	2	379	120	0.37 (0.17;0.56)	90.2%	0.001
Microscopy, RDT and PCR	3	631	194	0.29 (0.14;0.45)	94.6%	< 0.001

Geographically, the majority of studies (n = 19, 76%) were conducted in Southern Nigeria [14, 19–36], while six studies (24%) were conducted in Northern Nigeria [37–42]. Regarding diagnostic methods, microscopy was the most frequently employed technique, used in 15 studies (60%) as the sole diagnostic method. PCR alone was used in one study (4%), while RDTs alone were employed in three studies (12%). Combined diagnostic approaches were utilized in six studies: one study (4%) used both microscopy and RDT, two studies (8%) employed both microscopy and PCR, and three studies (12%) utilized all three methods (microscopy, RDT, and PCR). For the subgroup meta-analysis by diagnostic method, only diagnostic categories with at least two studies were included. Therefore, studies using PCR alone (n = 1) and those combining microscopy with RDT (n = 1) were excluded from the subgroup analysis.

All included studies employed a cross-sectional design. The temporal distribution showed that 24 studies (96%) were conducted after 2010, reflecting contemporary malaria epidemiology in Nigeria.

### Overall prevalence of asymptomatic malaria

**Fig. 2:**
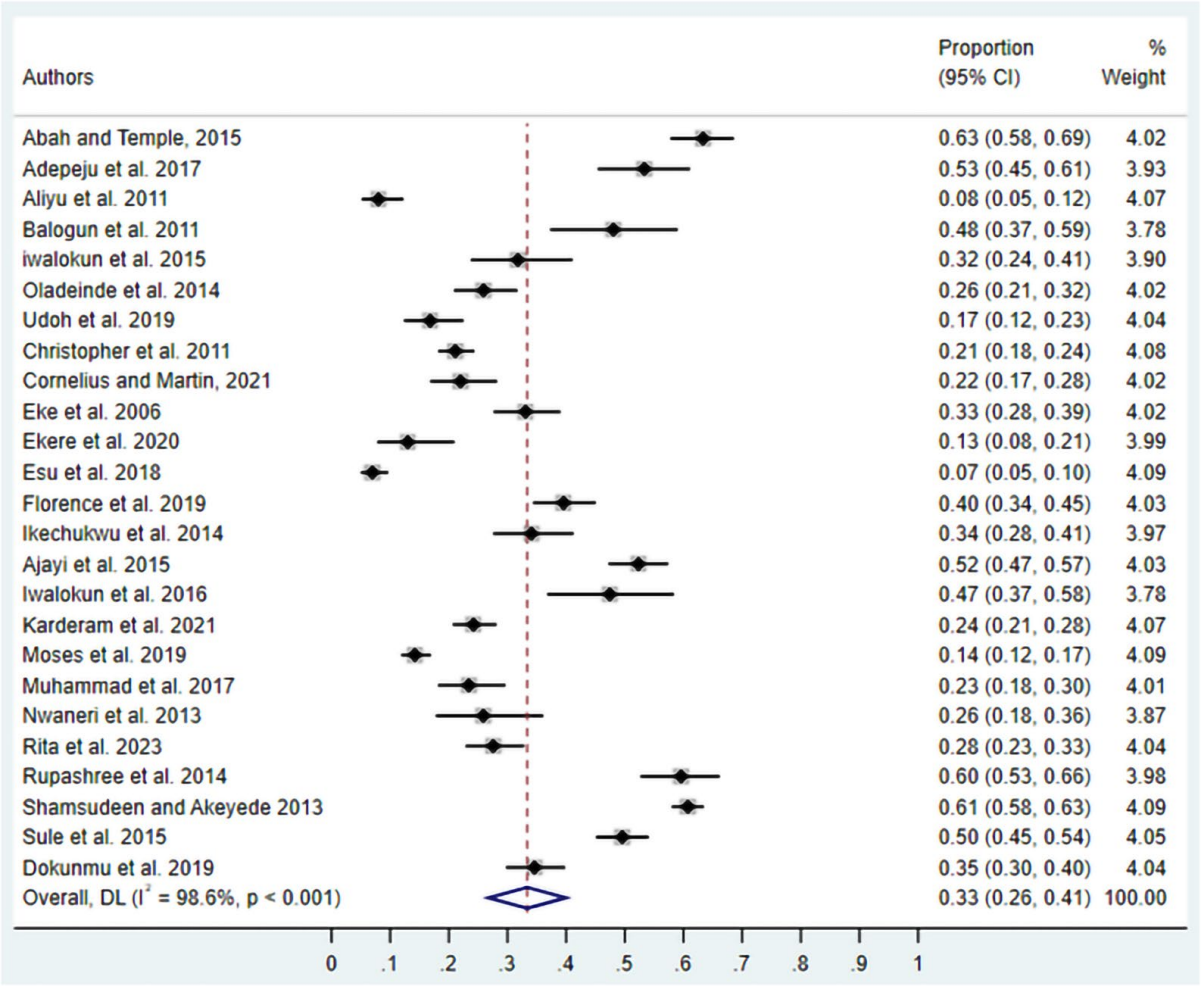
Forest plot of pooled prevalence of asymptomatic malaria in Nigeria. Individual study estimates are presented chronologically with 95% CIs. The diamond at the bottom represents the overall pooled prevalence estimate. Study weights are shown as percentages. random-effects model

The random-effects meta-analysis of 25 studies revealed a substantial pooled prevalence of asymptomatic malaria in Nigeria of 33% (95% CI 26–41%) (Fig. 2). The analysis demonstrated significant heterogeneity across studies (I^2^ = 98.6%, P < 0.001), indicating considerable variability in prevalence estimates between individual studies, which justified the use of subgroup analyses to explore potential sources of this heterogeneity.

### Subgroup analysis by study population

**Fig. 3:**
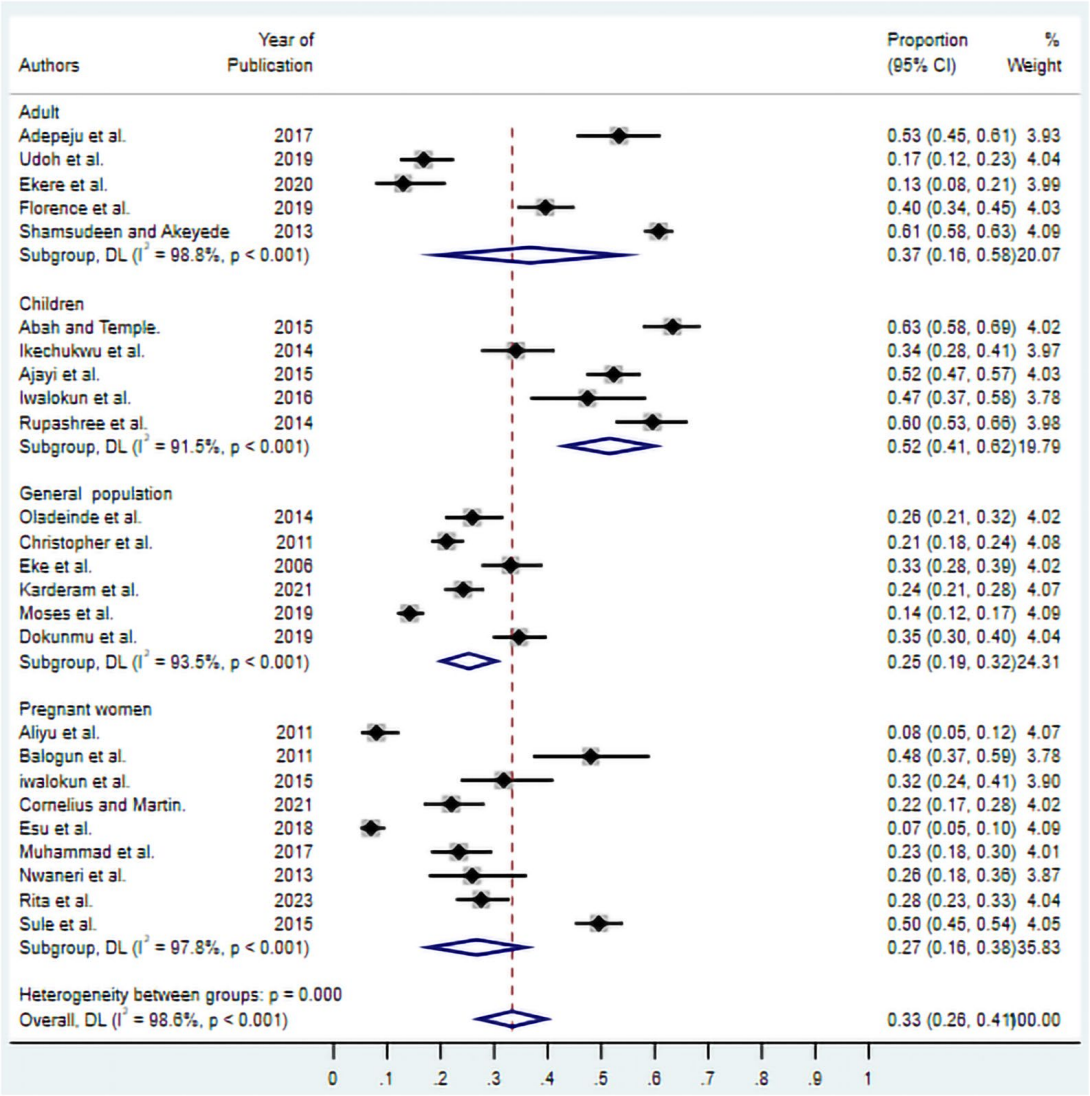
Subgroup analysis of asymptomatic malaria prevalence by in Nigeria study population. Studies are stratified by adults, children, the general population, and pregnant women. The diamonds represent subgroup pooled estimates with 95% CIs. Individual study estimates are shown as squares with horizontal lines representing 95% CIs. Study weights are presented as percentages. random-effects model

Subgroup analysis by target population revealed notable differences in asymptomatic malaria prevalence across demographic groups (Fig. 3). Children exhibited the highest burden of asymptomatic malaria with a pooled prevalence of 52% (95% CI 41–62%), establishing them as the most vulnerable population group. Adults demonstrated a moderate prevalence of 37% (95% CI 16–58%), while pregnant women showed a prevalence of 27% (95% CI 16–38%). The general population had the lowest pooled prevalence at 25% (95% CI 19–32%). These findings highlight significant age-related variations in asymptomatic malaria burden, with paediatric populations bearing disproportionately higher infection rates.

### Subgroup analysis by geographic region

**Fig. 4:**
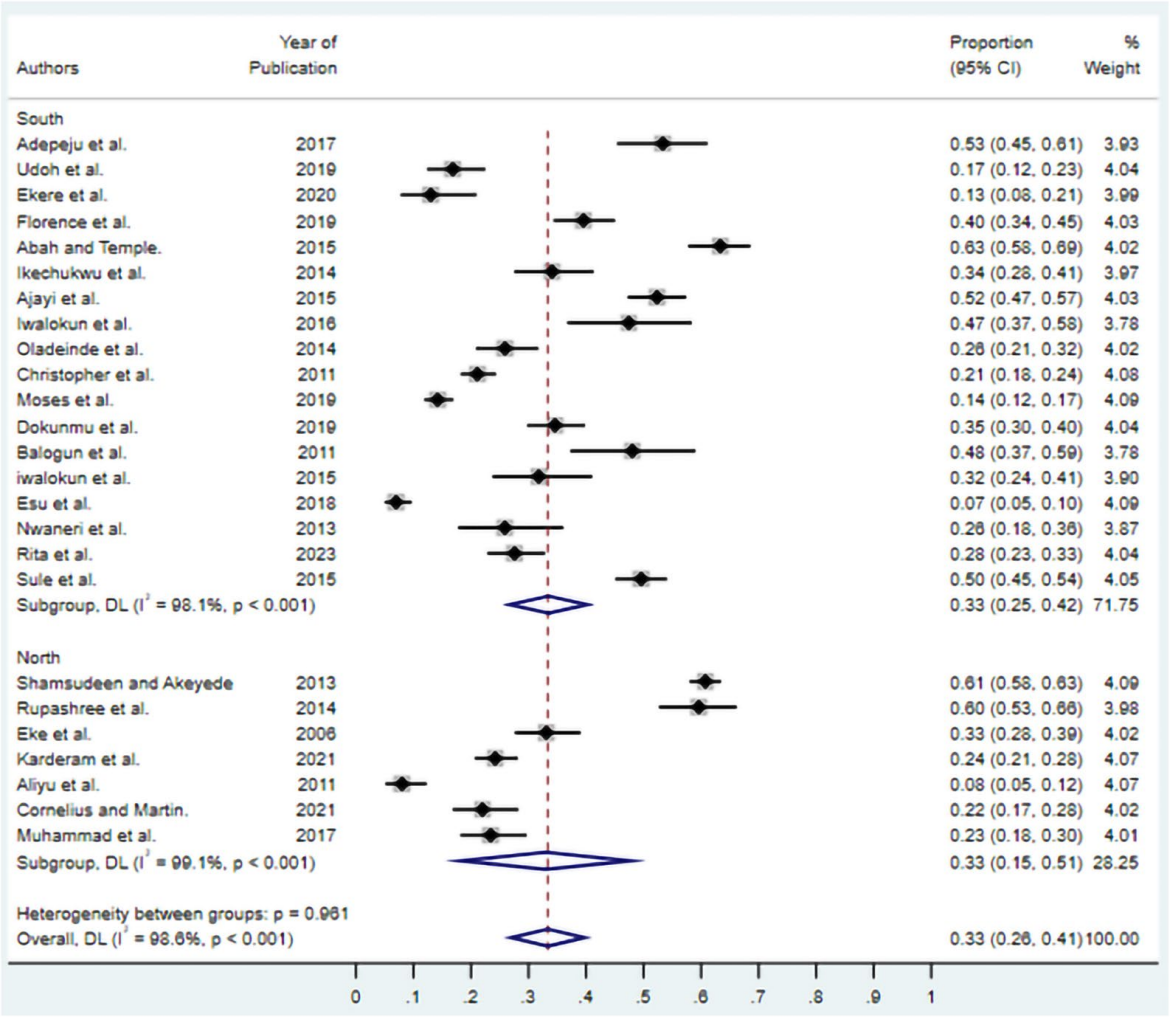
Subgroup analysis of asymptomatic malaria prevalence in Nigeria by geographic region. Studies are stratified by the South and North regions. The diamonds represent subgroup pooled estimates with 95% CIs. Individual study estimates are shown as squares with horizontal lines representing 95% CIs. Study weights are presented as percentages. random-effects model

Regional analysis revealed comparable asymptomatic malaria prevalence between Nigeria’s major geographic zones (Fig. 4). Southern Nigeria demonstrated a pooled prevalence of 33% (95% CI 26–41%), while Northern Nigeria showed an identical pooled prevalence of 33% (95% CI 13–53%). Despite similar point estimates, Northern Nigeria exhibited wider confidence intervals, likely reflecting the smaller number of studies conducted in this region and greater heterogeneity among the available data.

### Subgroup analysis by diagnostic method

**Fig. 5:**
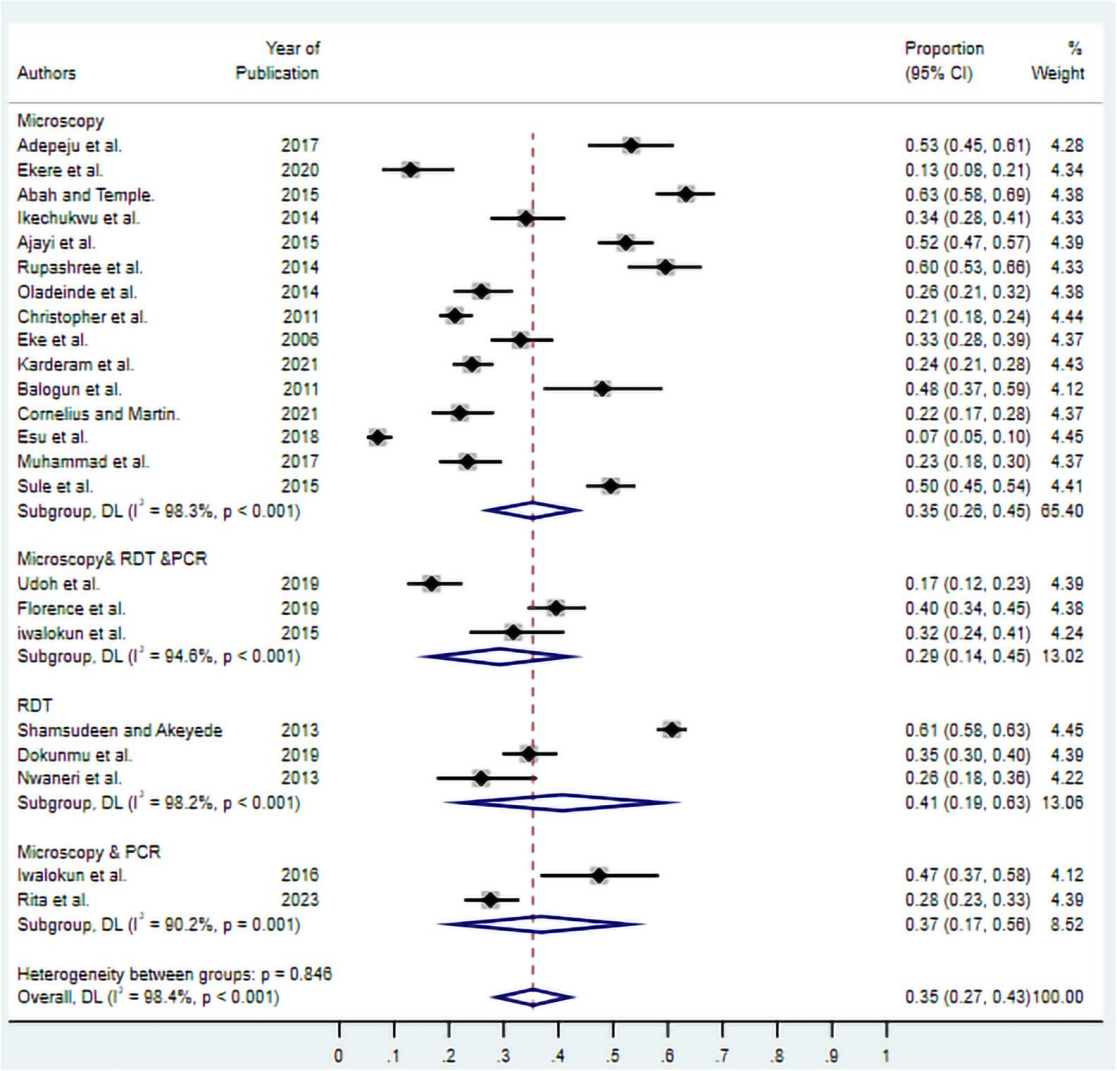
Subgroup analysis of asymptomatic malaria prevalence in Nigeria by diagnostic method. Studies are stratified by microscopy, microscopy & PCR, microscopy & RDT & PCR, and RDT. The diamonds represent subgroup pooled estimates with 95% CIs. Individual study estimates are shown as squares with horizontal lines representing 95% CIs. random-effects model

Diagnostic method significantly influenced prevalence estimates, with rapid diagnostic tests (RDTs) yielding the highest pooled prevalence of 41% (95% CI 19–63%) across three studies. Microscopy alone, the most commonly used diagnostic approach, produced a pooled prevalence of 35% (95% CI 26–45%). Studies employing combined microscopy and PCR methods estimated a prevalence of 37% (95% CI 17–56%) across two studies. The most conservative estimates were observed in studies using combined microscopy, RDT, and PCR approaches, which yielded a pooled prevalence of 29% (95% CI 14–45%) with reduced heterogeneity compared to other diagnostic categories (Fig. 5).

### Publication bias assessment

**Fig. 6:**
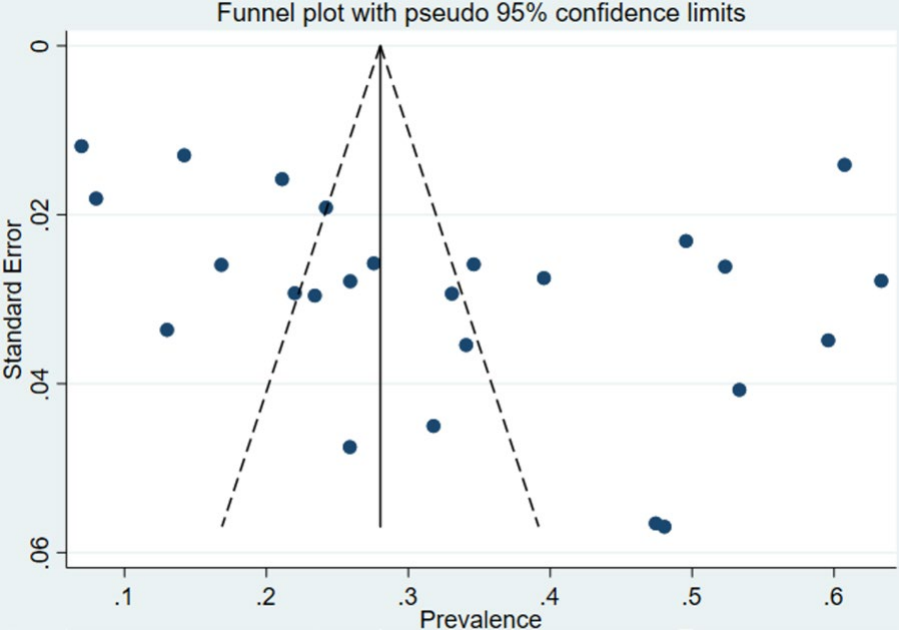
Funnel plot for the assessment of publication bias in the included studies. Each dot represents an individual study plotted against standard error (y-axis) and prevalence (x-axis). The vertical line represents the pooled estimate, and the diagonal lines represent the pseudo 95% confidence limits. Asymmetry suggests potential publication bias

Publication bias was evaluated using both statistical and visual methods. Egger’s regression test (Table 2) for small-study effects showed no statistically significant evidence of publication bias (bias coefficient: P = 0.093). Visual inspection of the funnel plot demonstrated a reasonably asymmetric distribution of studies around the pooled estimate, with studies distributed on both sides of the vertical reference line (Fig. 6). The combined statistical and visual assessments indicated no significant publication bias.

**Table 2:** Egger’s test statistics of the prevalence of malaria in Nigeria

Std_Eff	Coef	Std. Err	T	P >|t|	95%CI
Slope	0.134	0.091	1.46	0.157	− 0.05–0.32
Bias	6.993	3.989	1.75	0.093	− 1.26–15.25

### Meta-regression analysis

**Fig. 7:**
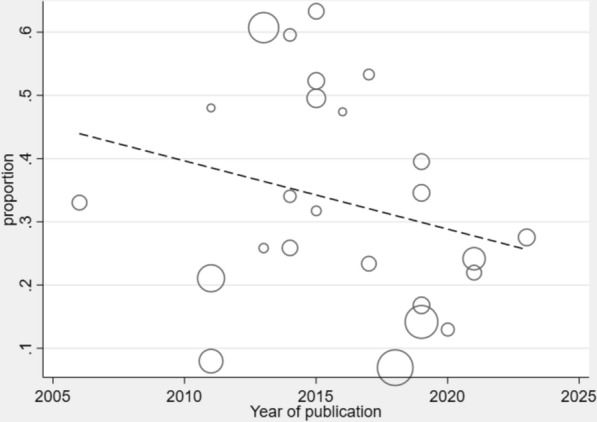
Meta-regression analysis of asymptomatic malaria prevalence in Nigeria by publication year. The analysis shows the temporal trend of asymptomatic malaria prevalence in Nigeria between 2005 and 2025. Each circle represents an individual study, with circle size proportional to study weight. The dashed line shows the regression line indicating a declining trend in prevalence over time. The y-axis shows the prevalence proportion, and the x-axis shows year of publication

Meta-regression was performed to explore sources of heterogeneity by examining the association between study publication year and prevalence estimates. The analysis revealed a statistically significant declining trend in asymptomatic malaria prevalence over time (Fig. 7). However, publication year explained only a modest proportion of the observed between-study heterogeneity (I^2^ = 98.6%), indicating that other study characteristics contribute substantially to the variability in prevalence estimates.

### Factors associated with asymptomatic malaria in Nigeria

**Fig. 8:**
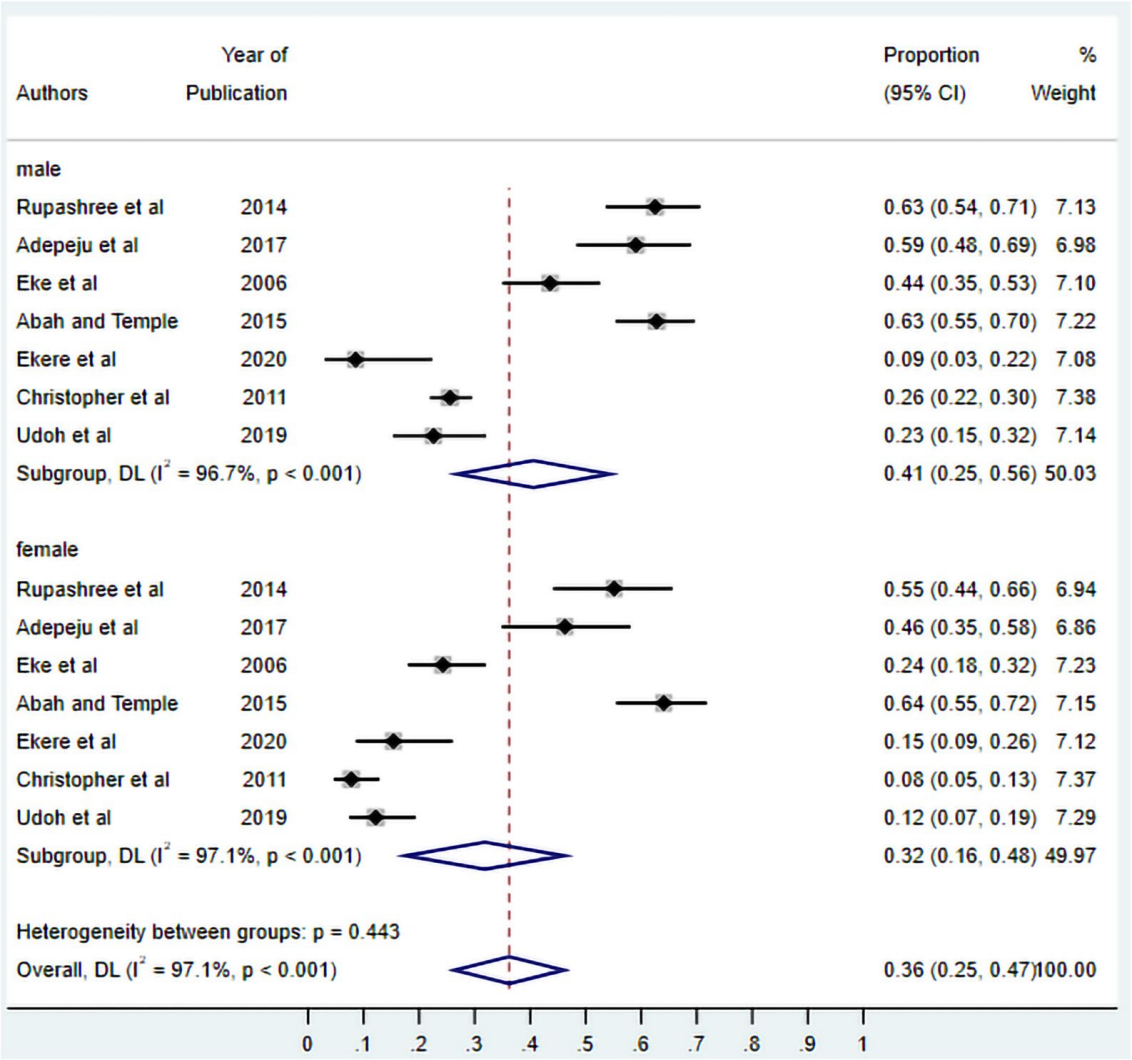
Forest plot of asymptomatic malaria prevalence in Nigeria by sex. Studies are stratified by male and female populations. The diamonds represent subgroup pooled estimates with 95% CIs. Individual study estimates are shown as squares with horizontal lines representing 95% CIs. Study weights are presented as percentages. random-effects model; I^2^, measure of heterogeneity

***Sex and asymptomatic malaria*:** There were seven studies identified that assessed the association between sex and asymptomatic malaria infection (Fig. 8). The Meta-analysis estimated the pooled prevalence of asymptomatic malaria at 41% (95% CI 0.25–0.56) among males, 28% (95% CI 0.15–0.47) among females, and 32% (95% CI 0.22–0.46) overall. While males exhibited a slightly higher prevalence, the difference was not statistically significant (p = 0.37). Substantial heterogeneity (I^2^ > 95%) was evident, reflecting variations in geographic settings, diagnostic approaches, and study populations, with prevalence ranging from 9 to 64%.

**Fig. 9:**
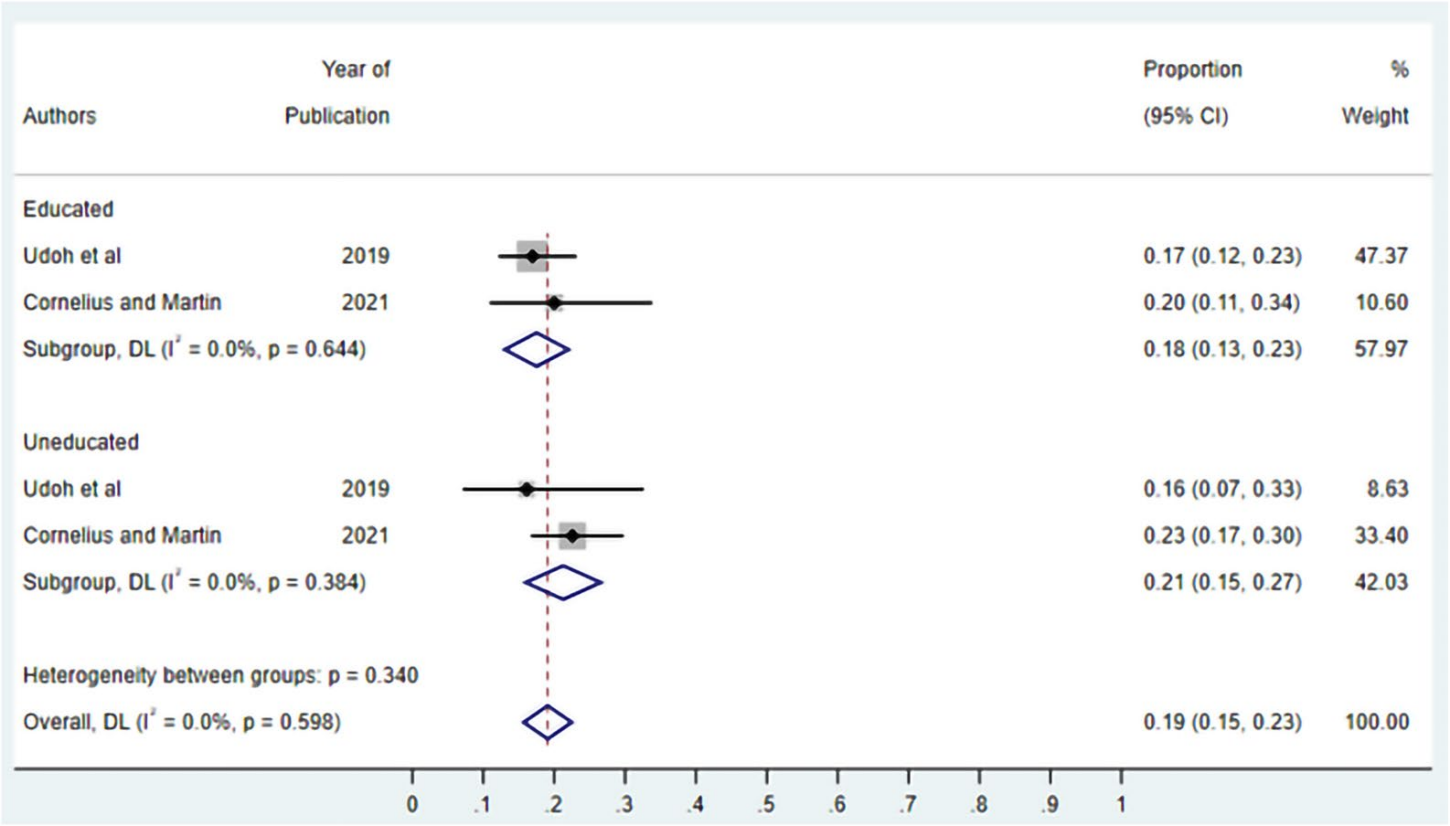
Forest plot of asymptomatic malaria prevalence in Nigeria by education status. Studies are grouped by educated and uneducated populations. The diamond represents the overall pooled estimate with 95% confidence intervals (CI). Individual study estimates are shown as squares with horizontal lines representing 95% CIs. The size of each square is proportional to the study weight. random-effects model; I^2^, measure of heterogeneity

***Education and asymptomatic malaria:*** Two studies were selected to determine the association between education and asymptomatic malaria infection (Fig. 9). The Meta-analysis evaluated the prevalence of asymptomatic malaria among individuals based on education status. Among those with education, the pooled prevalence was 18% (95% CI 0.13–0.23), while for those without education, the prevalence was slightly higher at 21% (95% CI 0.15–0.23), with no statistically significant difference (p = 0.38).

**Fig. 10:**
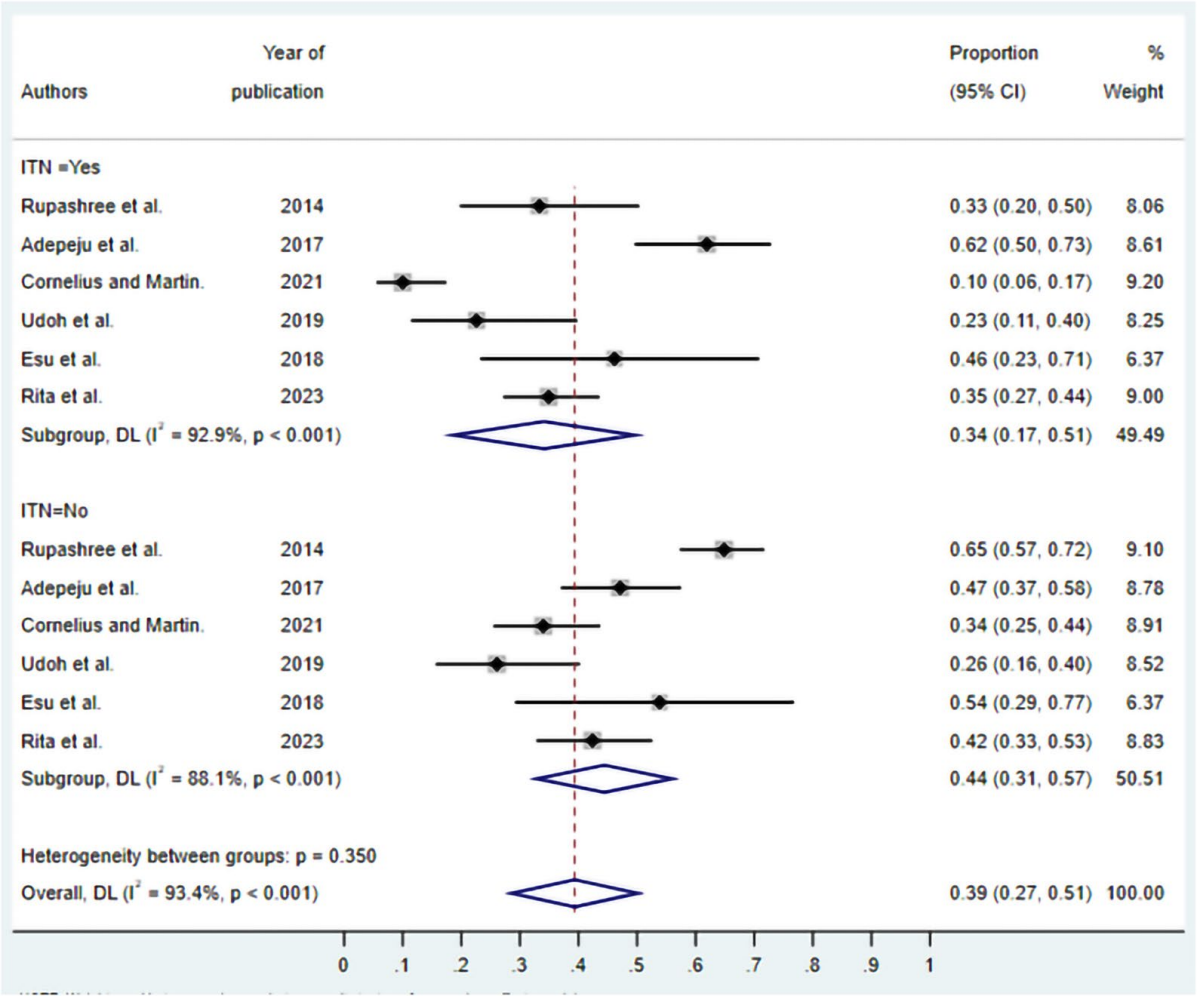
Forest plot of asymptomatic malaria prevalence in Nigeria stratified by insecticide-treated net (ITN) usage. Studies are grouped by ITN use (Yes/No). The diamond represents the overall pooled estimate with 95% CI. Individual study estimates are shown as squares with horizontal lines representing 95% CIs. random-effects model; I^2^, measure of heterogeneity

***ITN utilization and asymptomatic malaria:**** Seven* studies were selected to assess the association between ITN utilization and asymptomatic malaria infection (Fig. 10). The meta-analysis reveals that the pooled prevalence of asymptomatic malaria among ITN users is 34% (95% CI 0.1–0.51), while non-users have a higher pooled prevalence of 44% (95% CI 0.31–0.51). Substantial heterogeneity was observed in ITN users (I2 = 92.9%) and non-users (I2 = 88.1%), with a test for subgroup differences showing no statistically significant difference between groups (p = 0.350).

## Discussion

Malaria continues to pose a significant global public health burden, with sub-Saharan Africa experiencing disproportionately high transmission rates. Nigeria faces significant obstacles in malaria control and elimination, with the high burden of asymptomatic infections reflecting intense transmission [43]. This persistent exposure promotes partial immunity, allowing many infections to remain undetected while continuing to sustain transmission. Asymptomatic *Plasmodium* infections are characterized by the presence of parasites in individuals who remain clinically asymptomatic, creating a hidden reservoir that perpetuates community-level transmission cycles while evading routine detection and treatment protocols [44, 45].

This systematic review and meta-analysis determined a pooled prevalence of asymptomatic malaria in Nigeria of 33% (95% CI 0.26–0.41). The prevalence estimate observed in this analysis demonstrates consistency with previous systematic reviews and meta-analyses, including a study of asymptomatic malaria infection among pregnant women in sub-Saharan Africa (26.1%) [46] and a Nigeria-specific analysis (34.3%) [16]. However, the current pooled prevalence exceeds that reported in systematic reviews of studies conducted in Ethiopia among asymptomatic populations (6.7%) [47] and in children, when considering sub-Saharan Africa in general (25%) [44].

Individual primary studies across Africa demonstrate considerable heterogeneity in prevalence estimates, with rates ranging from 5.1% among migrant farmworkers in Ethiopia [48] to 41.4% in Burkina Faso [49] and 41.87% in Cameroon [50]. Comparable studies conducted in Uganda and Ghana report prevalence estimates between 24 and 28% [51, 52]. This substantial variation in prevalence estimates can be attributed to multiple epidemiological and methodological factors, including differences in malaria transmission intensity, diverse ecological and climatic parameters (temperature, precipitation patterns, and altitude), variations in diagnostic methodologies, heterogeneous study designs, distinct target populations, and varying effectiveness of local malaria control interventions.

Meta-regression analysis revealed a statistically significant declining temporal trend in asymptomatic malaria prevalence across the study period (2005–2025). The regression analysis demonstrated a negative association between publication year and prevalence estimates, with more recent studies consistently reporting lower prevalence rates compared to earlier investigations. This finding is consistent with the Nigeria Malaria Indicator Survey [53], which documented substantial reductions in infection prevalence compared to a decade earlier. This temporal pattern suggests that asymptomatic malaria burden in Nigeria may have declined over the past two decades, potentially reflecting the cumulative impact of intensified malaria control interventions, enhanced case management protocols, and evolving transmission dynamics.

The diagnostic methodology employed significantly influences prevalence estimation outcomes in malaria surveillance studies. RDTs demonstrated the highest pooled prevalence at 41%, despite their recognized limitations in detecting low-density parasitaemia characteristic of asymptomatic infections. This elevated prevalence can be attributed to the prolonged circulation of HRP2 antigens, which persist in the bloodstream for several weeks following parasite clearance, consequently generating false-positive results and inflating prevalence estimates [54]. Multiple studies indicate that HRP2-based rapid diagnostic tests (RDTs) show reduced sensitivity for detecting asymptomatic malaria in adults, largely because parasite densities in this group are typically very low. For instance, research from Angola found that several infections confirmed as HRP2-positive by antigen assays were not detected by standard RDTs due to parasite concentrations of only about 3–5 parasites/µL [55]. In contrast, PCR technology offers superior analytical sensitivity and accuracy for detecting submicroscopic infections [56]. However, PCR remains significantly underutilized in routine surveillance programmes due to substantial barriers, including high implementation costs, complex logistical requirements, and the need for specialized technical expertise. These constraints limit its widespread adoption, particularly in resource-limited settings where malaria burden is typically highest.

These findings highlight a critical gap in current diagnostic capabilities and underscore the pressing need for the development of affordable, field-deployable diagnostic tools that maintain high sensitivity for detecting asymptomatic infections. Such innovations are particularly crucial for improving surveillance accuracy in rural and underserved populations, where the true burden of asymptomatic malaria remains largely unmeasured and consequently inadequately addressed by current public health interventions.

In this study, children ≤ 15 years emerged as the most at-risk group with a prevalence of 52%. This is consistent with prior reports across sub-Saharan Africa, where school-aged children are recognized as significant reservoirs of infection [51]. This result highlights that younger individuals are more vulnerable due to immature immune responses and continuous exposure. This vulnerability could be linked to inadequate malaria control measures or insufficient monitoring and assessment of malaria control programmes. The development of immunity is slow and requires repeated exposure to the *Plasmodium* parasite by the *Anopheles* mosquito vector. Additionally, protection from the disease seems to decline without continuous exposure. The rate at which natural immunity is acquired is largely influenced by the intensity of transmission and the age of the individual [57]. Interestingly, a study in Ghana reported a higher prevalence among male school children aged 12–14 years [52], suggesting the role of age- and sex-specific behavioural and immunological factors. Collectively, these findings highlight the need for targeted interventions in school-aged populations, who may inadvertently perpetuate transmission.

The meta-analysis assessment for publication bias revealed mixed findings. While the funnel plot demonstrated visual asymmetry suggesting potential bias, Egger’s regression test yielded a non-significant result (P = 0.093), indicating insufficient statistical evidence to confirm publication bias among the included studies. These findings suggest that the meta-analysis results are unlikely to be substantially influenced by preferential publication of studies reporting significant or positive outcomes.

Long-lasting insecticidal nets (LLINs) represent a fundamental component of malaria prevention strategies, effectively reducing human-vector contact and diminishing overall disease burden [48]. However, in the analysis, asymptomatic malaria prevalence was generally higher among individuals not using ITNs compared to ITN users, indicating a protective trend. However, this association did not reach statistical significance, likely due to the limited number of studies, variability in study quality, and modest sample sizes. Notably, higher infection rates were observed among males, potentially attributable to increased nocturnal outdoor activities and less use of prevention tools like ITN that enhance vector exposure risk. Additionally, educational attainment demonstrated no statistically significant association with asymptomatic malaria prevalence. These findings underscore the critical importance of comprehensive community-wide health education programmes that can empower individuals to adopt evidence-based preventive behaviours and actively participate in integrated malaria control strategies, including optimal LLIN utilization and indoor residual spraying (IRS) implementation.

The substantial prevalence of asymptomatic malaria documented in this study carries profound implications for malaria control and elimination strategies in Nigeria. Asymptomatic carriers, while remaining clinically silent, function as persistent transmission reservoirs that sustain community-level parasite circulation, even in areas with high intervention coverage. Traditional surveillance systems that focus exclusively on symptomatic cases risk significantly underestimating true infection burden, particularly in high-transmission settings where a substantial proportion of the population may harbour asymptomatic infections at any given time. Consequently, strengthening surveillance to better capture asymptomatic infections is essential. In high-transmission settings such as Nigeria, this may be achieved through population-based surveys (e.g., DHS, MIS), sentinel surveillance, or targeted studies among high-risk groups such as school-age children and pregnant women, rather than routine systematic screening [8] Furthermore, the development and widespread deployment of cost-effective diagnostic technologies like highly sensitive rapid diagnostic test capable of detecting low-level parasitaemia are crucial for achieving accurate case detection and monitoring progress toward elimination objectives.

Community engagement strategies, targeted behaviour change communication, and comprehensive educational campaigns must be prioritized to reduce stigma associated with malaria, enhance ITN and IRS uptake, and reinforce public understanding of the epidemiological significance of asymptomatic infections in sustaining malaria transmission. These integrated approaches are fundamental to achieving sustainable reductions in malaria burden and advancing toward elimination goals in Nigeria.

## Conclusion

This meta-analysis reveals that asymptomatic malaria prevalence in Nigeria mirrors the trends observed in other endemic sub-Saharan African countries. While higher prevalence may be seen in certain sub-populations (e.g., school-aged children), the consistent and high prevalence across regions of Nigeria calls for comprehensive, geographically inclusive strategies. Strengthening proven malaria control strategies, including universal ITN coverage and use, improved access to case management, seasonal malaria chemoprevention for children, and expanded intermittent preventive treatment in pregnancy, remains the cornerstone for reducing Nigeria’s malaria burden. Once transmission has been substantially lowered through these approaches, targeted strategies such as focal screening and treatment may complement existing interventions.

## Supplementary Information

Additional file 1.

## Data Availability

No datasets were generated or analysed during the current study.

## Abbreviations

RDT: Rapid Diagnostic Test
PCR: Polymerase Chain Reaction
IRS: Indoor Residual Spray
ITN: Insecticide Treated Net
LLIN: Long Lasting Insecticide net
CI: Confidence Interval
NOS: Newcastle-Ottawa Scale
PRISMA: Preferred Reporting Items for Systematic Reviews and Meta-Analyses
ACT: Artemisinin-Based Combination Therapy
PROSPERO: Preferred Register for Systematic Review and Meta-analysis
WHO: World Health Organization
