# Supplementary material for: Prevalence and associated risk factors of asymptomatic malaria in Nigeria: a systematic review and meta-analysis
Source: Malar J. 2025 Dec 31;25:71. doi: 10.1186/s12936-025-05671-5 (PMC12866038; doi:10.1186/s12936-025-05671-5)
Supplement: Supplementary file 1 — Additional file 1. [file 12936_2025_5671_MOESM1_ESM.docx]

**Title: Prevalence and Associated Risk Factors of Asymptomatic Malaria in Nigeria: A Systematic Review and Meta-Analysis.**

**Authors:** Alameen Mukhtar^1,2,3^, Mubarak Ismail^1,2,3^, Murtala Bindawa Isah^1,2,3*^, Aminu Usman^2^, Ismail Ayoade Odetokun^4^, Mohammed Auwal Ibrahim^5^, Abdulmalik Abdullahi Salman^5^, Hafsat Garba Bawa-Sani^6^, Shafique Sani Nass^7^, Baba Waru Goni^3^, Muhammad Nazir Shehu^8^, and Xiaoying Zhang^1^.

**Journal:** Malaria journal

**Content**

Supplementary Table S1 & S2 Quality Assessment of Included Studies on Asymptomatic Malaria Using the Newcastle-Ottawa Scale (NOS)

Table S1: Quality assessment of included studies using the Newcastle-Ottawa Scale

| **Study ID** | **Author (Year)** | **Poor** | **Fair** | **Good** |
| --- | --- | --- | --- | --- |
| 1 | Abah and Temple, 2015 | poor |  |  |
| 2 | Adepeju et al. 2017 |  | Fair |  |
| 3 | Aliyu et al. 2011 | Poor |  |  |
| 4 | Balogun et al. 2011 |  | fair |  |
| 5 | Iwalokun et al. 2015 |  | fair |  |
| 6 | Oladeinde et al. 2014 |  | Fair |  |
| 7 | Udoh et al. 2019 |  | Fair |  |
| 8 | Christopher et al. 2011 |  | Fair |  |
| 9 | Cornelius and Martin, 2021 |  | Fair |  |
| 10 | Eke et al. 2006 |  | Fair |  |
| 11 | Ekere et al. 2020 |  | Fair |  |
| 12 | Esu et al. 2018 |  | Fair |  |
| 13 | Florence et al. 2019 |  | fair |  |
| 14 | Ikechukwu et al. 2014 |  | Fair |  |
| 15 | Ajayi et al. 2015 |  | fair |  |
| 16 | Iwalokun et al. 2016 |  |  | Good |
| 17 | Karderam et al. 2021 |  | Fair |  |
| 18 | Moses et al. 2019 | Poor |  |  |
| 19 | Mohammed et al. 2017 |  | Fair |  |
| 20 | Nwaneri et al. 2013 |  |  | Good |
| 21 | Rita et al. 2023 |  | Fair |  |
| 22 | Rupashree et al. 2014 |  | Fair |  |
| 23 | Shamsudeen and Akeyede, 2013 |  | Fair |  |
| 24 | Sule et al. 2015 |  | Fair |  |
| 25 | Dokunmu et al. 2019 |  | Fair |  |

**Grade system**

Poor = (0-3)

Fair = (4-6)

Good = (7-9)

**NEWCASTLE - OTTAWA QUALITY ASSESSMENT SCALE**

**(adapted for cross-sectional studies)**

Table S2: Quality assessment of included studies using the Newcastle-Ottawa Scale

| Study ID | Author (Year) | Selection (0-4) | Comparability (0-2) | Outcome/ Exposure (0-3) | Total (0-9) |
| --- | --- | --- | --- | --- | --- |
| 1 | Abah and Temple, 2015 | 2 | 0 | 1 | 3/9 |
| 2 | Adepeju et al. 2017 | 2 | 0 | 2 | 4/9 |
| 3 | Aliyu et al. 2011 | 2 | 0 | 1 | 3/9 |
| 4 | Balogun et al. 2011 | 2 | 1 | 2 | 5/9 |
| 5 | Iwalokun et al. 2015 | 2 | 1 | 2 | 5/9 |
| 6 | Oladeinde et al. 2014 | 2 | 0 | 2 | 4/9 |
| 7 | Udoh et al. 2019 | 3 | 0 | 2 | 5/9 |
| 8 | Christopher et al. 2011 | 2 | 1 | 2 | 5/9 |
| 9 | Cornelius and Martin, 2021 | 2 | 1 | 2 | 5/9 |
| 10 | Eke et al. 2006 | 4 | 0 | 1 | 5/9 |
| 11 | Ekere et al. 2020 | 3 | 0 | 2 | 5/9 |
| 12 | Esu et al. 2018 | 3 | 0 | 2 | 5/9 |
| 13 | Florence et al. 2019 | 2 | 0 | 2 | 4/9 |
| 14 | Ikechukwu et al. 2014 | 3 | 1 | 2 | 6/9 |
| 15 | Ajayi et al. 2015 | 2 | 0 | 2 | 4/9 |
| 16 | Iwalokun et al. 2016 | 4 | 1 | 2 | 7/9 |
| 17 | Karderam et al. 2021 | 4 | 0 | 2 | 6/9 |
| 18 | Moses et al. 2019 | 2 | 0 | 1 | 3/9 |
| 19 | Mohammed et al. 2017 | 3 | 0 | 2 | 5/9 |
| 20 | Nwaneri et al. 2013 | 4 | 1 | 2 | 7/9 |
| 21 | Rita et al. 2023 | 2 | 0 | 2 | 4/9 |
| 22 | Rupashree et al. 2014 | 3 | 0 | 2 | 5/9 |
| 23 | Shamsudeen and Akeyede, 2013 | 3 | 0 | 2 | 5/9 |
| 24 | Sule et al. 2015 | 3 | 0 | 2 | 5/9 |
| 25 | Dokunmu et al. 2019 | 3 | 0 | 2 | 5/9 |

***Key Considerations*:**

**Selection (Max 4)**:

Representativeness, sample size justification, non-response handling, and exposure ascertainment.

**Comparability (Max 2)**:

Control for confounders (e.g., age, socioeconomic status).

**Outcome (Max 3)**:

Assessment method, statistical rigor, follow-up (if applicable).

Table S3: Data extraction sheet

| **Data Extraction** | | | | | | |
| --- | --- | --- | --- | --- | --- | --- |
| **Authors** | **Study State** | **Region** | **Study Design** | **Sample size** | **Cases** | **Diagnostic method** |
| Abah and Temple, 2015 | Bayelsa | South | Cross sectional | 300 | 190 | Microscopy |
| Adepeju et al. 2017 | Ondo | South | Cross sectional | 150 | 80 | Microscopy |
| Aliyu et al. 2011 | Sokoto | North | Cross sectional | 225 | 18 | Microscopy &RDT |
| Balogun et al. 2011 | Oyo | South | Cross sectional | 77 | 37 | Microscopy |
| Iwalokun et al. 2015 | Lagos | South | Cross sectional | 107 | 34 | Microscopy& RDT &PCR |
| Oladeinde et al. 2014 | Edo | South | Cross sectional | 247 | 64 | Microscopy |
| Udoh et al. 2019 | Lagos | South | Cross sectional | 208 | 35 | Microscopy& RDT &PCR |
| Christopher et al. 2011 | Osun | South | Cross sectional | 668 | 141 | microscopy |
| Cornelius and Martin, 2021 | Kogi | North | Cross sectional | 200 | 44 | Microscopy |
| Eke et al. 2006 | Abia State | South | Cross sectional | 257 | 85 | Microscopy |
| Ekere et al. 2020 | Cross River | South | Cross sectional | 100 | 13 | Microscopy |
| Esu et al. 2018 | Cross River | South | Cross sectional | 459 | 32 | Microscopy |
| Florence et al. 2019 | Lagos | South | Cross sectional | 316 | 125 | Microscopy& RDT &PCR |
| Ikechukwu et al. 2014 | Edo | South | Cross sectional | 179 | 61 | Microscopy |
| Ajayi et al. 2015 | Oyo | South | Cross sectional | 365 | 191 | Microscopy |
| Iwalokun et al. 2016 | Lagos | South | Cross sectional | 78 | 37 | Microscopy & PCR |
| Karderam et al. 2021 | Kaduna | North | Cross sectional | 500 | 121 | Microscopy |
| Moses et al. 2019 | Anambra | South | Cross sectional | 725 | 103 | PCR |
| Muhammad et al. 2017 | Sokoto | North | Cross sectional | 205 | 48 | Microscopy |
| Nwaneri et al. 2013 | Ondo | South | Cross sectional | 85 | 22 | RDT |
| Rita et al. 2023 | Osun | South | Cross sectional | 301 | 83 | Microscopy & PCR |
| Rupashree et al. 2014 | Kebbi | North | Cross sectional | 198 | 118 | Microscopy |
| Shamsudeen and Akeyede 2013 | Nasarawa | North | Cross sectional | 1200 | 729 | RDT |
| Sule et al. 2015 | Ogun | South | Cross sectional | 468 | 232 | Microscopy |
| Dokunmu et al. 2019 | ogun | South | Cross sectional | 338 | 117 | PCR &RDT |
